# Aversion to light is associated with impulsivity

**DOI:** 10.3389/fpsyg.2024.1352320

**Published:** 2024-08-14

**Authors:** Alicia C. Lander, Andrew J. K. Phillips, Elise M. McGlashan, Sean W. Cain

**Affiliations:** ^1^Flinders Health and Medical Research Institute (Sleep Health), Flinders University, Bedford Park, SA, Australia; ^2^School of Psychological Sciences, Monash University, Melbourne, VIC, Australia; ^3^School of Psychological Sciences, University of Melbourne, Melbourne, VIC, Australia

**Keywords:** light aversion, impulsivity, ipRGCs, circadian rhythms, light sensitivity

## Abstract

Exposure to bright light can be visually aversive. This study explored the association between light aversion and various facets of impulsivity. A total of 1,245 participants completed the UPPS-Impulsive Behavior Scale to assess five facets of impulsivity. Additionally, participants responded to questions regarding their aversion to light (e.g., how aversive do you find bright light?). Spearman’s correlation coefficients (rho) revealed that individuals who find light physically aversive, or who experience a negative physical response to exposure (e.g., nausea or headache) triggered by bright indoor light or sunlight, tend to act impulsively under extreme negative and positive affect. Individuals who experience a negative physical response to exposure display greater premeditation, indicating a higher likelihood of considering the potential consequences of their actions. Moreover, these individuals score lower on sensation-seeking, suggesting a reduced inclination to seek out thrilling or novel experiences. These results reveal a complex relationship between light aversion and impulsivity, where those who find light aversive tend to be less impulsive in general, but more impulsive under extreme positive or negative affect.

## 1 Introduction

In addition to enabling vision, light has important non-visual inputs to the brain. These non-visual inputs come from intrinsically photosensitive retinal ganglion cells (ipRGCs), which are a small collection of retinal cells which contain the photopigment melanopsin (Berson et al., 2002). ipRGCs project to areas of the brain associated with cognition and the reward system (e.g., the locus coeruleus and the lateral habenula) (Luo and Aston-Jones, 2009). Additionally, ipRGCs project to areas of the brain that are involved in the propagation and modulation of pain [e.g., the periaqueductal grey (PAG)] (Mokhtar and Singh, 2020). Although the effects of light on alertness and arousal have been well studied, less is known about the impact of light on somatic functioning.

Bright light exposure can be visually aversive for some individuals. Aversive reactions may be mild (such as squinting when in a bright room), or more severe, whereby bright light exposure results in physical symptoms such as nausea or headaches. Light aversion is often associated with physical and mental health concerns. Approximately 80 to 90% of individuals who experience migraines also experience visual light sensitivity (Watson et al., 2009), while patients with attention deficit hyperactivity disorder (ADHD) report hypersensitivity to light (Kooij and Bijlenga, 2014). In addition to ocular discomfort, ADHD tends to be associated with cognitive deficits experienced in the monitoring of attentional resources, including impulsivity, a key characteristic of ADHD (Wilens and Spencer, 2010). Given the projection of ipRGCs to brain areas involved in both reward regulation and cognition, and in pain regulation, it is plausible that altered non-visual light processing has both somatic and cognitive impacts, including potential effects on impulsivity.

Impulsivity is characterized as the tendency to act with little forethought or control (Burnett Heyes et al., 2012). Impulsivity is often associated with poor sleep quality, since personality traits that are associated with impulsivity are believed to increase vulnerability to insomnia and daytime impairments [e.g., urgency and (lack of) premeditation] (Roth, 2007; Schmidt et al., 2008). As ipRGCs directly project to areas of the brain associated with impulsivity and pain modulation, it is likely that impulsivity is associated with light aversion. Increased sensitivity of the circadian system to light has been associated with mental health disorders that have impulsivity as a key characteristic, including ADHD and bipolar disorder. Patients with bipolar disorder or people with related traits show hypersensitivity to light, which may result in the misalignment of rhythms and may contribute to dysregulation of mood and reward-related cognition (Hallam et al., 2009; Bullock et al., 2019). Therefore, the impulsivity seen in bipolar disorder and ADHD could be in part driven by an increase in the effects of light on associated brain regions, and individuals who tend to be more impulsive may experience greater aversion to light. In this study, we sought to test the hypothesis that facets of impulsivity were positively associated with aspects of light aversion.

## 2 Method

### 2.1 Participants

A total of 1,245 participants were recruited from the general community and completed an online questionnaire. Participants were recruited online via social media posts and Facebook advertisements (targeted to individuals over 18 years of age). Participants from anywhere in the world were eligible to participate in the study. Participants were aged between 18 and 65 years (*M*_age_ = 39.00, SD_Age_ = 14.11), with the majority of the participants being female (73%), Caucasian (80%), full-time workers (30%) and from Australia (65%). Participants were largely classified as morning types on the Morningness-Eveningness questionnaire (Horne and Ostberg, 1976; 70% morning types, *M* = 50.53, *SD* = 13.31). Participants followed a web link to an online survey via Qualtrics (Qualtrics, Provo, UT), where responses were collected between 2020 and 2022. Participants were offered the opportunity to be entered into a draw to win a $100AUD gift card, with one gift card awarded per ~100 participants. The study was approved by the Monash University Human Research Ethics Committee.

### 2.2 Materials

The Urgency, Premeditation, Perseverance, Sensation-Seeking-Positive Urgency (UPPS-P) Impulsive Behavior Scale (short-form version) (Cyders et al., 2014) was used to measure impulsivity. The UPPS-P is a self-report measure designed to assess the five facets of impulsivity: lack of perseverance, lack of premeditation, positive and negative urgency, and sensation-seeking. Negative and positive urgency refer to the tendency to act rashly under conditions of extreme negative and positive affect, respectively. Premeditation (lack of) assesses the tendency to delay action as a result of careful thinking and planning, while perseverance (lack of) refers to an individual’s ability to complete a task and avoid boredom. Lastly, sensation-seeking refers to the tendency to seek thrilling and exciting experiences. The UPPS-P is a valid and reliable self-report measure, with an internal consistency between 0.74 and 0.88 across the subscales (Cyders et al., 2014). Participants completed 5-questions assessing their individual physical aversion to light: (1) “How aversive do you find bright light (artificial light or sunlight)?” (2) “Does bright indoor/fluorescent light make you nauseated?” (3) “Does bright sunlight make you nauseated?” (4) “Does bright indoor/fluorescent light give you a headache?” (5) “Does bright sunlight give you a headache?.” The first item was a four-point Likert-type scale, with response options ranging from very aversive to not at all aversive. Items two to five were on a five-point Likert-type scale, with response options ranging from always to never. These questions were developed based on face validity.

### 2.3 Data analysis

All statistical analyses were performed using R Statistical Software (v4.3.1; Team, 2023). The alpha level for statistical significance was set at 0.05 for all analyses. To examine the relationship between facets of impulsivity and light aversion, a partial Spearman’s rank correlation was conducted. The correlation was conducted using the PResidual package in R (Liu et al., 2020), which computes the partial Spearman’s rank correlation coefficient (rho) between our variables (e.g., light aversion and positive urgency), while adjusting for sex and age (Liu et al., 2020). This approach involves fitting five specific models of our light aversion variables on our covariates (sex and age), five specific models of our impulsivity variables on our covariates (sex and age), obtaining the probability-scale residuals from all models, and then calculating their correlation coefficient (rho) resulting in 25 correlations. To adjust for multiple comparisons, corrections were made using the Bonferroni-Holm method (Holm, 1979) for each individual predictor (i.e., the subscales of the UPPS-P Impulsive Behavior Scale). Adjusted *p*-values are presented, and an alpha level of 0.05 was applied.

## 3 Results

**Figure 1:**
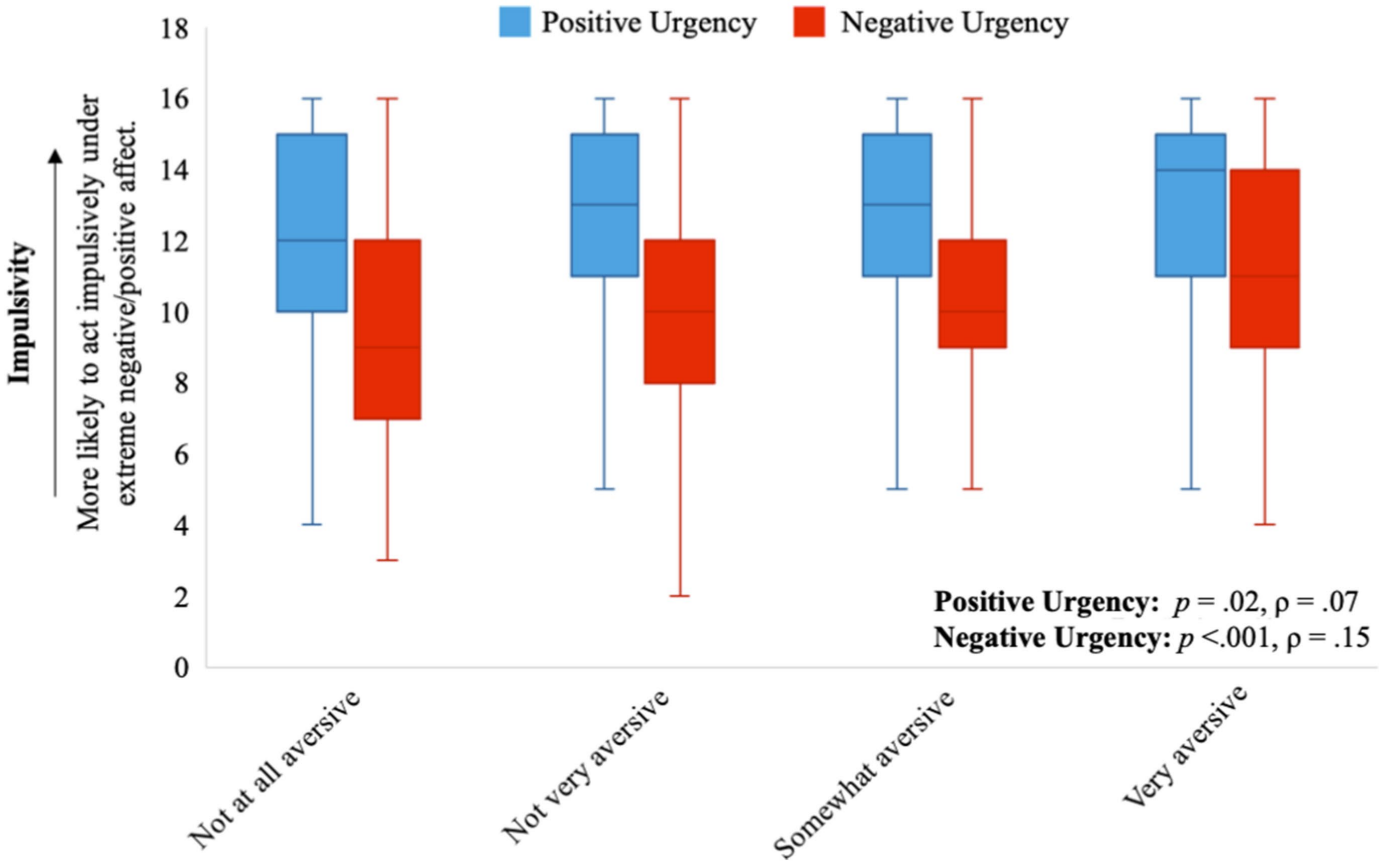
The distribution of traits of impulsivity (positive and negative urgency) for each level of overall light sensitivity. Positive urgency is represented by the orange box plots and negative urgency is represented by the blue box plots.

**Figure 2:**
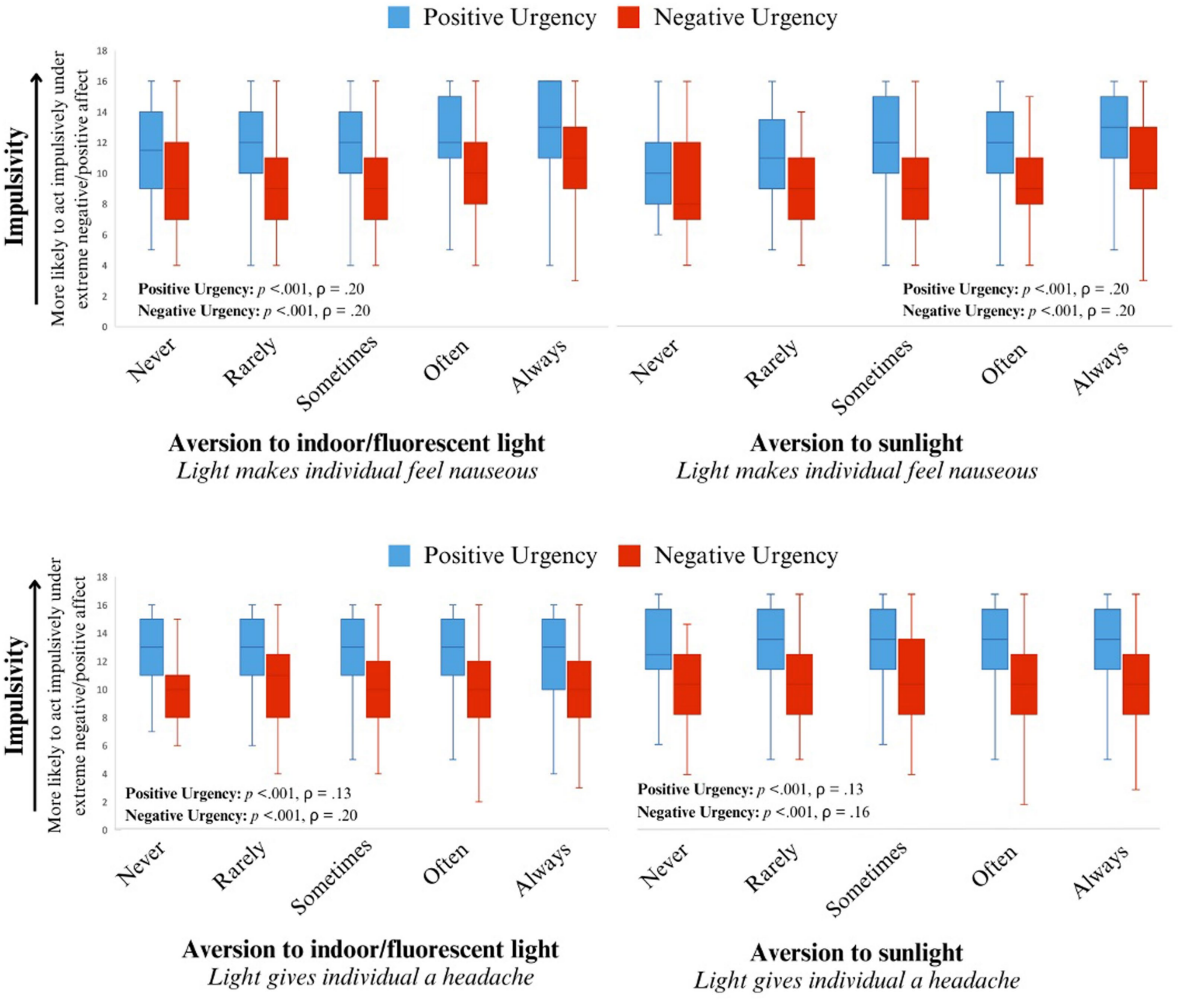
The distribution of traits of impulsivity (positive and negative urgency) for each level of light sensitivity resulting in individuals experiencing nausea **(top)** and headache **(bottom)**. Positive urgency is represented by the orange box plots and negative urgency is represented by the blue box plots.

**Figure 3:**
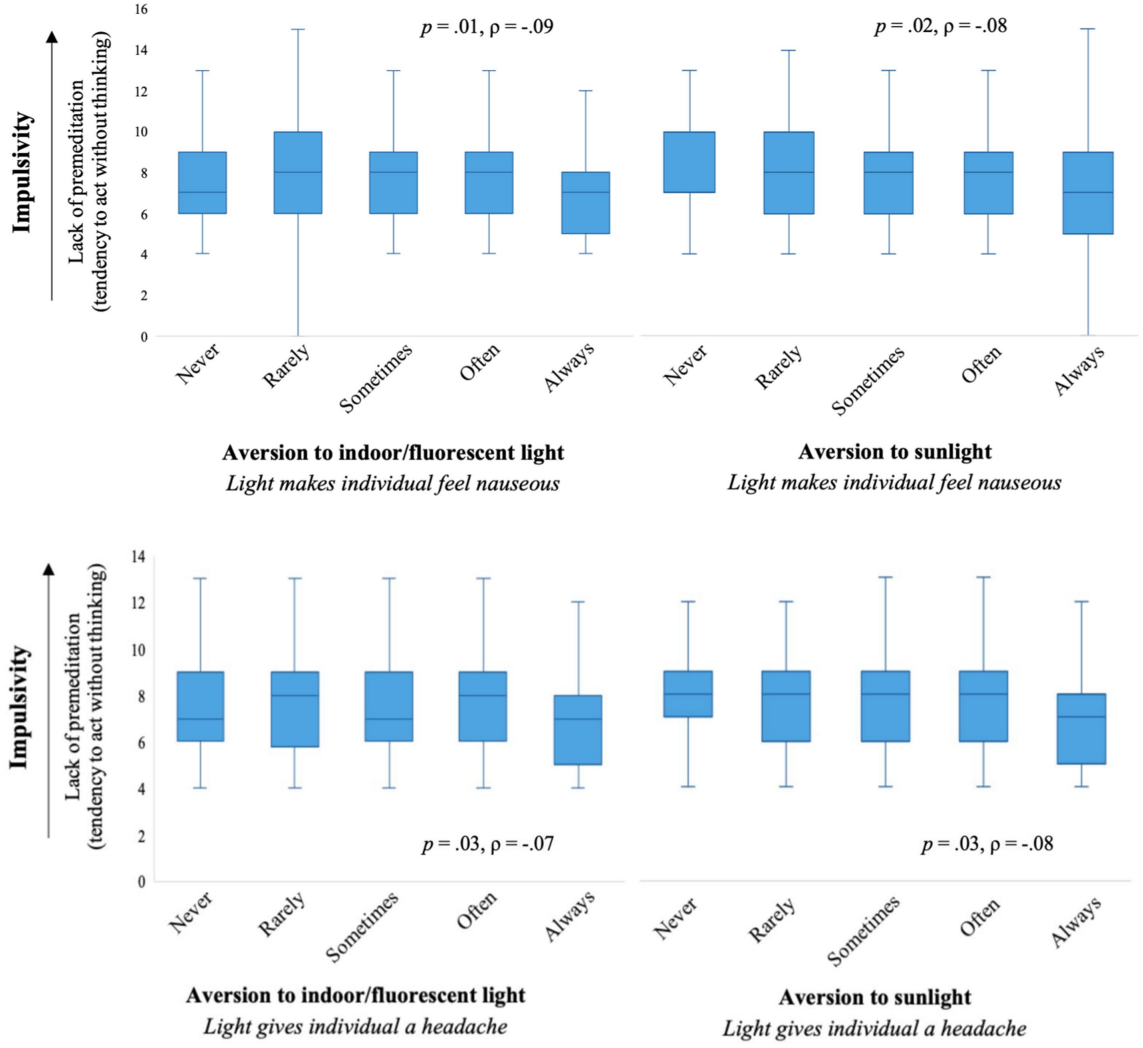
The distribution of premeditation (lack of) for each level of light sensitivity resulting in an individual experiencing nausea **(top)** or getting a headache **(bottom)**.

While adjusting for sex and age, two facets of impulsivity, positive and negative urgency, correlated positively and significantly with several aspects of light aversion. Individuals who found light physically aversive tended to report acting impulsively under both extreme negative affect (ρ = 0.15, *p* < 0.001) and positive affect (ρ = 0.07, *p* = 0.02). These findings were consistent with the unadjusted Spearman’s rank correlation (see Supplementary Table S1). Individuals who found bright indoor light and/or sunlight made them nausea or gave them a headache tended to act impulsively under extreme negative and positive affect (all *p* < 0.001; see Table 1 for Spearman’s correlations and Figures 1–3 for visualization of the data). The Spearman’s rho for indoor light and/or sunlight giving an individual a headache was higher than the unadjusted Spearman’s rank correlation. Alternatively, the Spearman’s rho for indoor light and/or sunlight making an individual nauseous was lower than the unadjusted Spearman’s rank correlation, suggesting that part of these correlations may be explained by their association with sex or age.

**Table 1:** Correlations between light aversion and positive and negative urgency, controlling for age and sex.

	Positive urgency		Negative urgency		Premeditation (lack of)		Perseverance (lack of)		Sensation seeking	
	Rho (ρ)	*p*	Rho (ρ)	*p*	Rho (ρ)	*p*	Rho (ρ)	*p*	Rho (ρ)	*p*
Aversive	0.07	**0.02**	0.15	**<0.001**	−0.01	0.69	0.005	0.99	−0.04	0.76
Nausea										
Indoor light	0.20	**<0.001**	0.20	**<0.001**	−0.09	**0.01**	−0.001	0.99	−0.01	0.99
Sunlight	0.20	**<0.001**	0.20	**<0.001**	−0.08	**0.02**	−0.01	0.99	−0.02	0.99
Headache										
Indoor light	0.13	**<0.001**	0.20	**<0.001**	−0.07	**0.03**	0.04	0.77	−0.03	0.80
Sunlight	0.13	**<0.001**	0.16	**<0.001**	−0.08	**0.03**	−0.02	0.99	−0.09	**0.004**

Bold values indicate significance (*p* < .05).

Perseverance (lack of) did not significantly correlate with any aspects of light aversion (all *p* > 0.05). Premeditation (lack of) did not significantly correlate with finding light aversive; however, individuals who found bright indoor light and/or sunlight made them nauseous or gave them a headache had greater premeditation, meaning they were more likely to act with consideration of the potential consequences of their behavior (all *p* < 0.001; see Figure 3). These findings were lower than the unadjusted Spearman’s rank correlation, except for the association between sunlight making an individual nauseous and premeditation which was consistent with the unadjusted Spearman’s rank correlation. Lastly, the facet of sensation-seeking did not significantly correlate with most aspects of light aversion (*p* > 0.05), except for individuals who found sunlight gave them a headache as they had lower sensation seeking (*p* = 0.001). See Table 1 for the Spearman’s correlations.

## 4 Discussion

This study examined the relationship between light aversion and facets of impulsivity. While previous clinical work has focused on physiological markers of light sensitivity (e.g., pupillary, hormonal markers), we studied the relationship between facets of impulsivity and aversive somatic responses to light. We found a significant association between traits of impulsivity and light aversion. Specifically, we found that individuals who find bright light to be physically aversive, or who find bright indoor light and sunlight makes them nauseous, tend to act more impulsively under both extreme negative and positive affect. We also found that individuals who find bright indoor light and/or sunlight makes them nauseous or gives them a headache exhibit more premeditation, thus are more likely to consider the consequences of their behavior before engaging in them. These results suggest a potential association between light sensitivity and impulsivity, specifically highlighting that when physically averse to bright light, individuals are more likely to act impulsively under extreme positive and negative valence but are more likely to exhibit cognitive control.

Exposure to light can be overwhelming for individuals who find it aversive, sometimes leading to uncomfortable reactions like nausea and headaches. Our findings reveal a connection between sensory discomfort in light sensitive individuals and increased impulsivity during intense positive and negative emotions. This heightened emotional state disrupts rational decision-making and heightens distractibility, with positive emotions fostering undue optimism concerning possible outcomes of situations, and negative urgency driving impulsive behavior in hope to reduce the negative emotion (Smith and Cyders, 2016). Individuals with heightened sensitivity to sensory stimuli, such as sunlight, may be more susceptible to adverse psychological symptoms due to the inclination to process information more deeply, leading to sensory overload and extreme affect (Brindle et al., 2015). It is plausible that the negative affect associated with the sensory discomfort is mediated by emotion regulation. Exposure to light may compromise emotional regulation strategies in light-averse individuals, hindering their ability to adapt to situational demands (Brindle et al., 2015). Consequently, their difficulty in effectively regulating emotions may explain their increased tendency toward impulsive behaviors. Previous research has shown that impulsive behavior in response to intense negative affect is associated with poor emotion regulation, including heightened rumination and a reduced use of reappraisal and perspective-taking strategies (Johnson et al., 2020). Therefore, our findings suggest that our results are due to light sensitive individuals exhibiting poor emotion regulation in response to extreme affect, leading to impulsive behaviors as a means of coping with the intensity of negative emotions.

Individuals reporting discomfort or headaches in response to bright light tend to display an enhanced inclination towards premeditative thinking. Light averse individuals demonstrate a propensity for deliberating and evaluating potential outcomes before engaging in behaviors, which may be partly attributed to the interconnected functioning of the amygdala and ventromedial prefrontal cortex (vmPFC). The vmPFC plays a key role in rational decision-making, while the amygdala is primarily associated with emotional processing (Kroker et al., 2022). Exposure to light appears to strengthen the connection between the amygdala and vmPFC (McGlashan et al., 2021), with a potential for this connection to be more pronounced in individuals with heightened light sensitivity. Consequently, when light averse individuals are exposed to light, this intensified amygdala-vmPFC connectivity (and potentially other PFC enhanced connections yet unknown) may contribute to heightened cognitive control, characterized by increased premeditation. Furthermore, this heightened connectivity may activate the behavioral inhibition system, which reduces behavioral responses in an effort to avoid adverse consequences (Xu et al., 2021). The activation of a more vigilant behavioral inhibition system is theorized to be linked to individuals with sensory processing sensitivity, as they tend to exercise greater caution in their behavior (Acevedo et al., 2023), thus fostering increased conscientiousness and a deeper cognitive processing of information, ultimately shaping their decision-making process. It would be beneficial for future investigations to utilize functional magnetic resonance imaging (fMRI) to examine the amygdala-vmPFC connection in individuals with heightened sensitivity to light, and its impact on cognitive control.

In addition to projecting to brain regions associated with impulsivity and light aversion, ipRGCs also project to areas involved in pain modulation and sensory processing. These parallel pathways might underlie the relationship between physical aversion to light and impulsivity. The thalamic nuclei, which receive input from the spinal trigeminal system responsible for relaying sensory modalities (Dolgonos et al., 2011), may further exacerbate aversive reactions to light. Furthermore, ipRGCs project to the periaqueductal grey (PAG), a key component in the propagation and modulation of pain (Mokhtar and Singh, 2020), and pain centers in the thalamus, an important locus for sensory processing and an area implicated in impulsivity (Moulton et al., 2009; Katz and Digre, 2016, p. 09). The projection of ipRGCs to brain regions associated with both impulsivity and pain modulation suggests a potential mechanism underlying the relationship. It is possible that individuals who are highly sensitive have greater amounts of light information being sent from the retina to pain centers, resulting in an aversive reaction. Additionally, this increased amount of light information might extend to other areas involved in impulsivity and reward regulation, resulting in a shared vulnerability between pain centers and impulsivity. Clinical groups characterized by impulsivity not only exhibit heightened sensitivity to light, but also present a heightened vulnerability to migraines (Mahmood et al., 1999; Ortiz et al., 2010). Individuals diagnosed with bipolar disorder or ADHD tend to exhibit increased sensitivity to light, including both retinal sensitivity, and measures which reflect activity in the circadian clock (Lewy et al., 2006; Hallam et al., 2009; Madsen et al., 2021). In addition to increased physiological sensitivity to light, these groups frequently experience migraines, with bipolar disorder having a prevalence rate as high as 39% (almost double the general population) (Mahmood et al., 1999; Ortiz et al., 2010). This suggests a shared vulnerability between aversion to light, pain modulation, and impulsivity.

Individuals averse to light, as they might be more prone to sensory overload, may organize their life and alter their behavior around minimizing their exposure to light. Therefore, individuals who recognize the nauseating effect of bright light, for example, may actively avoid brightly lit rooms or wear sunglasses for extended periods during the day to alleviate discomfort (Kooij and Bijlenga, 2014). However, minimizing exposure to sensory stimulation like light, particularly during the day, can have negative consequences for both physical and mental health. Daytime bright light exposure is associated with lower odds of several psychiatric conditions, and better self-reported mood and wellbeing (Golden et al., 2005; Burns et al., 2022, 2023). Additionally, daytime light exposure influences our circadian rhythms, leading to advanced timing of rhythms and enhanced circadian amplitude (Bano-Otalora et al., 2021). Therefore, light avoidant behavior not only renders individuals more vulnerable to psychiatric conditions such as bipolar disorder, where impulsivity is already a heightened risk, but also may disrupt an individual’s circadian rhythms by potentially delaying their rhythms and reducing circadian amplitude. Targeting the reduction in light sensitivity (e.g., specific optical tints) may allow light sensitive individuals to still benefit from daytime light exposure without experiencing the negative physiological effects.

It should be noted that this study had an overrepresentation of women. Men typically exhibit higher levels of impulsivity compared to women, specifically having higher scores for sensation seeking, indicating a propensity for seeking complex and intense experiences (Costa and McCrae, 2008). Additionally, men tend to demonstrate greater lack of perseverance, leading to difficulties in maintaining focus on a boring or mundane task (Costa and McCrae, 2008). On the other hand, women tend to score higher on the impulsiveness facet of the NEO Personality Inventory-Revises (NEO-PI-R), which is closely related to the facet of negative urgency (Costa and McCrae, 2008). These differences underscore significant variations in impulsivity between sexes. The relationship we observed between negative urgency and light aversion could be partly driven by higher levels of negative urgency in women. However, it should be noted that part of our findings remain consistent even after controlling for sex, which suggests that the observed relationships between positive urgency and light aversion is not solely driven by sex differences. Furthermore, as explained above, daytime light exposure influences our circadian rhythms and can lead to the advanced timing of rhythms and circadian amplitude. However, this study did not capture daily light exposure patterns and therefore future studies should include light exposure patterns to assess light exposure behavior and impulsivity. The cross-sectional design of our study does not allow for conclusions to be made regarding the directionality of our correlations, with future research required to determine the directionality of these variables. Overall, our findings show a relationship between light aversion, characterized by symptoms such as nausea and headache, and impulsivity in the context of extreme positive or negative affect. While existing literature has predominantly focused on physiological measures of light sensitivity, our study has shown relationships between subjective experiences of light aversion and impulsivity. This work expands the current understanding of how sensory experiences interact with psychological traits. Future research can build upon these findings to further examine relationships between psychological traits, subjective experiences, and physiological measures of light sensitivity to better characterize interactions between non-image forming light responses.

## Data availability statement

The raw data supporting the conclusions of this article will be made available by the authors, without undue reservation.

## Ethics statement

The studies involving humans were approved by Monash University Human Research Ethics Committee (Project #24512). The studies were conducted in accordance with the local legislation and institutional requirements. The participants provided their written informed consent to participate in this study.

## Author contributions

AL: Conceptualization, Data curation, Formal analysis, Investigation, Methodology, Project administration, Writing – original draft, Writing – review & editing. AP: Writing – review & editing. EM: Writing – review & editing, Supervision. SC: Supervision, Writing – review & editing.
