# Supplementary material for: Aversion to light is associated with impulsivity
Source: Front Psychol. 2024 Aug 14;15:1352320. doi: 10.3389/fpsyg.2024.1352320 (PMC11350512; doi:10.3389/fpsyg.2024.1352320)
Supplement: Supplementary file 1 [file Table_1.DOCX]

Supplementary Material

Aversion to light is associated with impulsivity

**Alicia C Lander^*^, Andrew JK Phillips, Elise M McGlashan and Sean W Cain**

*** Correspondence:** Corresponding Author: sean.cain@flinders.edu.au

# Supplementary Figures and Tables

**Table 1.** Unadjusted Spearman’s rank correlations between light aversion and positive and negative urgency

|  | Positive Urgency | | Negative Urgency | | Premeditation  (lack of) | | Perseverance  (lack of) | | Sensation  seeking | |
| --- | --- | --- | --- | --- | --- | --- | --- | --- | --- | --- |
|  | rho (ρ) | *p* | rho (ρ) | *p* | rho (ρ) | *p* | rho (ρ) | *p* | rho (ρ) | *p* |
| Aversive | .07 | **.02** | .15 | **<.001** | -.01 | .65 | .005 | .86 | -.04 | .21 |
| Nausea |  |  |  |  |  |  |  |  |  |  |
| Indoor light | .19 | **<.001** | .20 | **<.001** | -.09 | **.001** | -.001 | .96 | -.01 | .79 |
| Sunlight | .20 | **<.001** | .19 | **<.001** | -.08 | **.003** | -.01 | .71 | -.02 | .54 |
| Headache |  |  |  |  |  |  |  |  |  |  |
| Indoor light | .13 | **<.001** | .21 | **<.001** | -.07 | **.01** | .04 | .15 | -.03 | .22 |
| Sunlight | .13 | **<.001** | .17 | **<.001** | -.08 | **.01** | -.02 | .55 | -.09 | **.001** |
